# Assessment of glycemic control among patients undergoing surgical treatment for advanced diabetic retinopathy in Saudi Arabia

**DOI:** 10.1186/s40942-026-00826-1

**Published:** 2026-03-02

**Authors:** Ahmed Radi Algethami, Thuraya Zaki Jastaniah, Jouri Khalid Futaini, Abeer Saleh Fallatah, Shahad Aref Almatrafi, Sally Farahat Elsehimy, Ali Mohammed Bin Mahfodh

**Affiliations:** 1 Department of Surgical Retina, Eye Health Center, King Abdullah Medical City, Makkah, Saudi Arabia; 2 College of Pharmacy-Umm Alqura University, Makkah, Saudi Arabia; 3 College of Applied Medical Sciences -Umm Alqura University, Makkah, Saudi Arabia

## Abstract

**Background:**

Glycemic Control and its most common complication Diabetic retinopathy are a leading cause of visual impairment among diabetic patients which has a significant effect on patients’ quality of life.

**Aim:**

This study aims to determine the impact of poor glycemic control (HbA1c) in preoperative tests and how it will affect the surgical outcome for advanced diabetic retinopathy.

**Methods:**

A retrospective case series study was employed at King Abdullah Medical City, Makkah. Analyzing 91 patients who underwent surgical treatment for advanced diabetic retinopathy from January 1, 2021, to 31 December 2021. Data were collected using the Track Care system, targeting patients whose preoperative level of glycemic control (HbA1c) was uncontrolled.

**Results:**

The final outcome determined that preoperative vision was poor in 18 patients with controlled HbA1c (≤7), and 52 patients (57%) had uncontrolled HbA1c (>7%). Compared with postoperative vision, it was poor in 20 patients with controlled HbA1c and 49 with uncontrolled HbA1c, and good in only 4 patients with controlled HbA1c and 18 with uncontrolled HbA1c (p>0.05). The average level of poorly controlled HbA1c in preoperative tests reaches 8.13 in our referred patients.

**Conclusion:**

This study concluded that the elevated HbA1c levels were associated with poor perioperative outcomes in diabetic patients undergoing surgical management for advanced retinopathy, highlighting the importance of optimizing glycemic control prior to surgery.

## Background

Diabetes mellitus (DM) is a chronic metabolic disorder characterized by persistently elevated blood glucose concentrations resulting from defects in insulin secretion, insulin action, or both. Several distinct forms of DM exist, including type 1 diabetes, type 2 diabetes, maturity-onset diabetes of the young (MODY), gestational diabetes, neonatal diabetes, and secondary diabetes associated with endocrine disorders, chronic steroid therapy, and other underlying conditions [1–3]. Worldwide estimates indicate that approximately one in every eleven adults is living with diabetes, with type 2 diabetes accounting for nearly 90% of all cases. The incidence of type 1 diabetes rises steadily from infancy, showing characteristic peaks between 4 and 6 years and again during early adolescence (10–14 years), with nearly 45% of affected children presenting before the age of ten [4].

Diabetes is associated with a spectrum of acute and chronic complications involving multiple organ systems. Persistent hyperglycemia contributes to microvascular and macrovascular injury, ultimately increasing the risk of cardiovascular disease, cerebrovascular events, peripheral neuropathy, diabetic kidney disease, and diabetic retinopathy [5–7].

Diabetic retinopathy is the most common retinal vascular complication of diabetes mellitus and a leading cause of visual impairment and blindness among working-age patients [8], with some studies reporting about 30% prevalence among the Saudi population [9, 10]. Diabetic retinopathy (DR) is a preventable complication of diabetes; however, it is a serious one, if not early recognized and properly managed, as it can lead to visual impairment. Unfortunately, most patients are asymptomatic until the very late stages. Consequently, the disease can rapidly progress before the initiation of therapy, leading to poor outcomes. The common risk factors for DR include hyperglycemia, dyslipidemia, hypertension, and long duration of diabetes. Therefore, it is important to determine possible preventive tools. In the prevention of diabetic retinopathy, it is essential to control glycemia, lipid profile and blood pressure [11]. It has been reported that the control of blood glucose level can delay or alleviate the development of diabetic retinopathy, nevertheless, hyperglycemia is a major risk factor for the development of diabetic retinopathy.

Although diabetic retinopathy used to lead to blindness in many cases, most of its complications are currently preventable and treatable, since the introduction of laser treatment photocoagulation and pars plana vitrectomy (PPV) and recent advances in Anti-vascular endothelial growth factor (VEGF) agent [12–14] Controlling the level of glucose in the blood is very important thus it can prevent the development of diabetic retinopathy. Patients with DR may resort to surgical operations in the advanced stages of the disease. These surgical interventions are prone to complications, and some might be affected by HbA1c levels. Previous reports have shown that complications related to surgical intervention can occur intraoperatively or postoperatively [7, 8]. It has been reported that bleeding after vitrectomy is more likely to occur in patients with high preoperative HbA1c levels and hyperglycemia just before vitrectomy, in addition to the duration of the operation may increase the risk of postoperative bleeding after vitrectomy in diabetic retinopathy patients [7–9].

However, the relationship between glycemic control and retinopathy progression is complex. While long-term glucose management is protective, several studies have reported that substantial or rapid reductions in blood glucose—particularly in patients with long-standing hyperglycemia—may trigger a transient, early worsening of DR. This paradoxical phenomenon underscores the delicate balance required during intensive glycemic correction and highlights the importance of individualized monitoring, especially for patients with advanced diseases [6].

Therefore, this study aims to report the glycemic control among diabetic patients with diabetic retinopathy who were referred for surgical interventions at King Abdullah Medical City in Makkah, Saudi Arabia.

## Methodology

### Study design

A retrospective study design was conducted between January 1, 2021, and 31 December 2022 of data collection. Ethical approval obtained from IRB in King Abdullah Medical City before study enrollment. The requirement for individual informed consent was formally waived by the Institutional Review Board/Ethics Committee.The study was conducted in accordance with the ethical principles outlined in the Declaration of Helsinki and relevant international ethical guidelines.

Human Ethics and Consent to Participate declarations: not applicable.

### Study setting and population

This study was performed for all patients who underwent surgical treatment for advanced diabetic retinopathy with all ages, genders, and nationalities. All patients’ records with a period from January 1, 2021, and 31 December 2022. Operative records were used to identify those who had been surgically repaired at King Abdullah medical city, Makkah at endocrinology or ophthalmology departments. While all incomplete patients’ records lack main data were excluded.

### Data collection and management

After setting the eligibility criteria for this study. Demographics, patients’ history, and clinical characteristics were considered in this study. Data was collected from patient’s medical records into electronic data collection forms not showing any nominative information at King Abdullah medical city, Makkah. All data was then extracted, analyzed using suitable analytical tests.

### Statistical analysis

Data was collected in an Excel sheet (Microsoft Office, 2021) and transferred to SPSS program version 27.0 for statistical analysis. Descriptive data was expressed in frequency and comparisons between demographic information and transfusion reactions were analyzed by ANOVA and unpaired t -test, The statistical significance was determined at a *p* value less than 0.05.

## Results

A total of 91 patients were included in the study, with a mean age of 41.6 ± 12.9 years. Of these, 46 (50.5%) were male, and 71 patients (78%) had Type 2 diabetes, while 20 patients (22%) had Type 1. Concerning glycemic control, 24 patients (26.4%) had controlled HbA1c (≤ 7), while 67 patients (73.6%) had uncontrolled HbA1c (> 7).

The distribution of HbA1c levels among the studied patients shows that the majority had suboptimal long-term glycemic control. Approximately 73.6% of participants exhibited HbA1c values > 7%. Only 26.4% achieved HbA1c values ≤ 7%.

The biochemical markers further support a metabolic profile consistent with elevated cardiovascular and renal risk. The mean serum creatinine level was 1.2 ± 0.32 mg/dL. Lipid parameters demonstrate significant dyslipidemia, with a notably elevated mean LDL cholesterol of 142.45 ± 36.29 mg/dL, exceeding recommended targets for diabetic patients. Meanwhile, the average HDL level of 51.01 ± 12.23 mg/dL falls within acceptable ranges, though the variation suggests that some individuals may still have suboptimal protective HDL levels. Triglyceride levels were also elevated, with a mean of 182.27 ± 67.88 mg/dL (Table 1).

**Table 1:** The main parameters reported among the study participants (*n* = 91)

Parameter	Frequency (%)
**HbA1c**	
≤ 7	24 (26.4%)
> 7	67 (73.6%)
**Biochemical markers**	**Mean ± S.D**
Serum creatinine	1.2 ± 0.32
LDL	142.45 ± 36.29
HDL	51.01 ± 12.23
Triglycerides	182.27 ± 67.88

Overall glycemic assessment among the reported participants was obtained among the biochemical data of the 91 patients demonstrates a clear pattern of poor metabolic control for fasting blood glucose (> 140 mg/dL), postprandial (> 180 mg/dL), and random blood glucose (> 200 mg/dL). Renal function, reflected by serum creatinine levels, ranges from normal to mildly elevated (0.93–1.72 mg/dL).

The lipid profile similarly reveals a high cardiometabolic burden. LDL cholesterol is elevated in a substantial proportion of patients (80–198 mg/dL). HDL levels show significant variability, with many patients having borderline or low protective levels (< 30 mg/dL). Triglyceride concentrations are also frequently elevated, consistent with atherogenic dyslipidemia typically seen in poorly controlled type 2 diabetes (84–256 mg/dL).

### Post-operative vision and glycemic control

The association between glycemic control and post-operative visual outcomes was not statistically significant (*p* = 0.317). Among controlled patients, 83.3% (20/24) had poor post-operative vision and 16.6% (4/24) had good vision. In the uncontrolled group, 73.1% (49/67) had poor vision while 26.9% (18/67) had good vision (Table 2).

**Table 2:** Correlation between Post-operative Vision and Glycemic Control

Post-operative vision	Glycemic control	
	Controlled	Uncontrolled
*Poor vision* (worse than 20/200 on the Snellen chart)	83.3 (20/24)	73.1 (49/67)
*Good vision*	16.6 (4/24)	26.9 (18/67)
**p-value**	0.317	0.416
**t/F value**	24.6	35.8

### Post-operative vision and pre-operative vision

A significant association was found between pre-operative and post-operative vision (*p* = 0.004). Patients with good pre-operative vision were more likely to maintain good post-operative vision (45.5% vs. 17.1%). Conversely, patients with poor pre-operative vision predominantly had poor post-operative outcomes (84.1%).

Analysis of systemic comorbidities showed no statistically significant associations with visual outcomes following treatment. Hypertension did not demonstrate a meaningful relationship with outcome status (*p* = 0.379); although poor outcomes were slightly more common among non-hypertensive patients (82.6%) compared with those who had hypertension (73.5%). Similarly, chronic kidney disease showed no significant association with postoperative visual results (*p* = 0.148). Patients with CKD exhibited a higher proportion of poor vision (88.9%) compared to those without CKD (72.6%), but this difference did not reach statistical significance.

Ischemic heart disease also failed to show a significant relationship with outcome status (*p* = 0.273). Poor outcomes were observed in 64.3% of patients with IHD, compared to 77.9% of those without the condition. Likewise, other systemic diseases demonstrated no significant association with visual prognosis (*p* = 0.142). Although poor vision was more frequently reported among patients with additional systemic conditions (85.7%) compared to those without (71.4%), these differences were not statistically significant (Table 3).

**Table 3:** ANOVA test for post-operative vision and systemic comorbidities

	Percentage (%)	t/F value	*p*-value
**Hypertension**	Poor outcomes	41.2	0.379
Yes	73.5		
No	82.6		
**Chronic Kidney Disease**	Poor vision	24.5	0.148
Yes	88.9		
No	72.6		
**Ischemic Heart Disease**	Poor outcomes	26.8	0.273
Yes	64.3		
No	77.9		
**Other systemic diseases**	Poor vision	34.8	0.142
Yes	85.7		
No	71.4		

## Discussion

This study aims to report the glycemic control among diabetic patients with diabetic retinopathy who were referred for surgical interventions at King Abdullah Medical City in Makkah, Saudi Arabia. In this study, a total of 91 patients were included in the study. Of these, 46 (50.5%) were male and 71 patients (78%) had Type 2 to indicate the high prevalence of T2DM than other types [15] and indicating the high prevalence of T2DM relative to other types.

The distribution of HbA1c levels among the studied patients shows that the majority had suboptimal long-term glycemic control. Approximately 73.6% of participants exhibited HbA1c values > 7%, indicating poor diabetes management and an increased risk of microvascular complications, including diabetic retinopathy. The low proportion reported in HbA1c values ≤ 7%, reflecting adequate glycemic control based on international guidelines, may indicate poor control in diabetes among the reported cases, which may lead to various complications such as DR [16].

The biochemical markers further support a metabolic profile consistent with elevated cardiovascular and renal risk. Serum creatinine level was at the upper limit of normal and may suggest early renal impairment in part of the cohort, particularly given the known association between diabetic nephropathy and retinopathy. Lipid parameters demonstrate significant dyslipidemia, with a notably elevated mean LDL cholesterol of for exceeding recommended targets for diabetic patients. Meanwhile, the average HDL level of 51.01 ± 12.23 mg/dL falls within acceptable ranges, though the variation suggests that some individuals may still have suboptimal protective HDL levels. Triglyceride levels were also elevated, highlighting an atherogenic lipid profile commonly observed in poorly controlled diabetes [17].

These findings indicate widespread uncontrolled diabetes, consistent with advanced disease severity and a high risk of microvascular complications. Only a minority of patients have glucose values near normal or borderline high, further emphasizing the predominance of hyperglycemia in this cohort [18].

Renal function, reflected by serum creatinine levels, ranges from normal to mildly elevated. Several patients have values approaching or exceeding the upper limit of normal, suggesting reduced renal filtration capacity or early diabetic nephropathy—an association commonly observed in individuals with longstanding diabetes and retinopathy [19].

The lipid profile similarly reveals a high cardiometabolic burden. LDL cholesterol is elevated in a substantial proportion of patients, often surpassing recommended therapeutic targets for individuals with diabetes. HDL levels show significant variability, with many patients having borderline or low protective levels. Triglyceride concentrations are also frequently elevated, consistent with atherogenic dyslipidemia typically seen in poorly controlled type 2 diabetes [17, 20].

Collectively, these findings describe a patient population characterized by poor glycemic control, dyslipidemia, and early renal functional changes, all of which contribute to the progression and severity of diabetic retinopathy. This metabolic profile is clinically relevant, particularly in patients undergoing surgical treatment for advanced stages of the disease, as these systemic abnormalities may influence disease progression, treatment outcomes, and postoperative recovery [21].

Overall, these findings reflect a patient population with suboptimal glycemic control and significant dyslipidemia, both of which are established contributors to the progression of diabetic retinopathy and may influence surgical outcomes in individuals undergoing treatment for advanced disease [18]. It is in agreement with Shawki et al. study, who demonstrated that patients with diabetes exhibited markedly elevated uric acid and creatinine levels, accompanied by significantly reduced HDL concentrations compared with the non-diabetic control group. A similar pattern was observed among individuals with diabetic retinopathy, who showed higher uric acid and creatinine and lower HDL levels than diabetic patients without retinopathy [22].

Across the whole cohort, 75.8% (69/91) had poor post-operative vision. The association between glycemic control and post-operative visual outcomes was not statistically significant (*p* = 0.317). Among controlled patients, 83.3% (20/24) had poor post-operative vision and 16.6% (4/24) had good vision. In the uncontrolled group, 73.1% (49/67) had poor vision while 26.9% (18/67) had good vision, which agrees with Schreur et al.‘s study, who reported that poor glycemic control is not correlated with poor postoperative vision as an outcome [23].

Analysis of systemic comorbidities showed no statistically significant associations (*p* > 0.05) with visual outcomes following treatment like hypertension or any ischemic heart diseases [24].

### Conclusion and recommendations

In this study, the referred patients had poor long-term glycemic control. Elevated HbA1c values are clinically important, as they are associated with increased perioperative risks and may adversely affect overall patient health, particularly in individuals undergoing surgical management for diabetic complications. These findings recommend and highlight the need for improved diabetes care and tighter metabolic control within our community. Enhancing the quality of diabetic management could help reduce surgical risks, mitigate disease progression, and ultimately improve patient outcomes. It has been suggested that the presence of systemic comorbidities, including hypertension, chronic kidney disease, ischemic heart disease, and other systemic illnesses, did not significantly influence the visual outcomes in this patient cohort.

## Data Availability

No datasets were generated or analysed during the current study.
